# Addison’s disease in a child: a case report

**DOI:** 10.1186/1687-9856-2015-S1-P40

**Published:** 2015-04-28

**Authors:** Nur Rochmah, Muhammad Faizi

**Affiliations:** 1 Faculty of Medicine-Airlangga University-dr Soetomo Hospital, Surabaya, East Java, Indonesia

## 

Addison's disease is chronic primary adrenal insufficiency, a rare disorder which is characterized by adrenocortical insufficiency. The sign and symptom in Addison's disease is nonspecific. This paper is to report Addison’s disease in a child focusing in diagnostic approach. Method is case report. Girl, 4 years old suffered from general weakness without paresthesia, fatigue, salt craving and hyperpigmentation including skin, lips, gum, buccal mucosa, hard palate and plantar creases. Tanner stage was prepubertal condition. Basal cortisol plasma level in the morning was 94.8 (50-250) µg/mL and 14.5 (25-125) µg/mL in the evening. This patient was performed ACTH stimulation test. The result revealed declining cortisol plasma level before (20.83) and 30’, 120’ after the test (16.53, 5.91, respectively). Free T4 was 17.31 (9-20) pmol/L; TSH 2.48 (0.25-5) uIU/mL. Adrenal ultrasound revealed no classification nor hemorrhage. Primary adrenal insufficiency was established. Tuberculosis is frequently reported in Addison. In our patient the Tuberculin skin test revealed negative. Treatment planned to be given were oral hydrocortisone 15 mg/m2 and oral fludrocortisone. Based on anamnesis, physical examination and laboratory findings, addison disease was established. As conclusion, beware of generalized weakness and hyperpigmentation in a child, it may be the symptom of adrenal insufficiency. Careful diagnosis procedure is very important.

Written informed consent was obtained from the patient for publication of this abstract and any accompanying images. A copy of the written consent is available for review by the Editor of this journal.

